# Effectiveness of a decision aid for colorectal cancer screening on components of informed choice according to educational attainment: A randomised controlled trial

**DOI:** 10.1371/journal.pone.0241703

**Published:** 2020-11-10

**Authors:** Pernille Gabel, Mette Bach Larsen, Adrian Edwards, Pia Kirkegaard, Berit Andersen

**Affiliations:** 1 Department of Public Health Programmes, Randers Regional Hospital, Randers, Denmark; 2 Division of Population Medicine, School of Medicine, Cardiff University, Cardiff, United Kingdom; 3 Department of Clinical Medicine, Aarhus University, Aarhus, Denmark; Griffith University, AUSTRALIA

## Abstract

**Background:**

The decision to take up colorectal cancer screening has to be made on informed grounds balancing benefits and harms. Self-administered decision aids can support citizens in making an informed choice. A self-administered web-based decision aid targeting citizens with lower educational attainment has been evaluated within the target population. However, the effectiveness in the general screening population remains unexplored. The aim of this study was to evaluate the effectiveness of a web-based decision aid for colorectal cancer screening on components of informed choice among previous non-participants in colorectal cancer screening.

**Methods and findings:**

The study was designed as a parallel randomised controlled trial among non-participants in colorectal cancer screening in Central Denmark Region (men and women aged 53–74 years). Respondents to baseline and follow-up questionnaires comprised the study population (n = 1,723). The intervention group received the decision aid electronically along with the second reminder. The control group received only the second reminder. The main outcomes (knowledge, attitudes, uptake and decisional conflict) were obtained through questionnaires data and from the Danish Colorectal Cancer Screening Database.

The decision aid increased the uptake rate by 8 percentage points (95% CI: 3.4;12.6) but had no effect on either knowledge (scale score differences: 0.09; 95% CI: -0.05;0.24) or attitudes (0.45; 95% CI: -0.00;0.91). Decisional conflict decreased by 1.69 scale points (95% CI: -3.18;-0.20). The effect was similar across educational attainment levels.

**Conclusions:**

The web-based decision aid offers a feasible way to provide individualised screening information in a "one size fits all" approach that may hold the potential to increase informed CRC screening uptake.

**Trial registration:**

ClinicalTrials.gov registration number: NCT03253888.

## Background

Colorectal cancer (CRC) screening using the fecal occult blood test (FOBT) reduces mortality from the disease [1] but there are also harms related to CRC screening, such as false negative and false positive screening results, over-diagnosis, over-treatment and risks of complications to colonoscopy. Therefore, taking up screening is a preference-sensitive choice [2, 3] that should be made on informed grounds.

There are three components of informed choice; knowledge, attitudes and behaviour. A choice based on relevant knowledge and with accordance between attitudes and actual behaviour is defined as informed [4, 5]. Studies have shown that only around 10% of citizens eligible for CRC screening make informed choices about participation in organised screening programmes in Australia and Germany [6, 7].

Decision aids are information materials designed to support informed decision-making by presenting benefits and harms about all available options, and supporting a genuine choice without coercion [8–10]. Self-administered decision aids are required in FOBT-based screening programmes where citizens usually decide about screening uptake without any contact with health care professionals. Self-administered decision aids can support citizens in making an informed choice about whether to take up CRC screening [6, 7, 11]. They have shown to increase knowledge [6, 7, 11–14] whereas they may induce less favourable attitudes towards screening [6, 7]. Results regarding screening uptake are inconclusive [6, 7, 11, 15].

A self-administered web-based decision aid [16] targeted at citizens with lower educational attainment, defined as level 1–2 according to the International Standard Classification of Education (ISCED 2011) [17], has been developed and evaluated among citizens with lower educational attainment [18, 19]. However, it is important to know the effect of providing the decision aid along with the reminder regardless of educational attainment levels to consider whether implementation in a CRC screening programme to all citizens might also be justifiable from the evidence.

Hence we evaluated the effectiveness of the web-based decision aid for CRC screening on the components of informed choice—knowledge, attitudes and uptake—among all educational attainment groups. We also assessed the effectiveness of the decision aid on decisional conflict.

## Methods

### Setting

The study was conducted in Central Denmark Region within the national CRC screening programme. The national Faecal Immunochemical Test (FIT)-based screening programme was introduced for citizens aged 50–74 years in 2014 with a four-year prevalence round were eligible citizens were invited once. It was fully implemented by the end of 2017 where after CRC screening is offered biennially. Citizens receive a letter comprising the invitation letter and a test-kit to collect a stool-sample, which is sent to the hospital for testing. Citizens, who do not return a stool sample within 45 days, receive a digital screening reminder. Non-participating citizens and citizens with a negative FIT-result are referred to the next screening round and receive a new screening invitation two years later. Citizens with a positive FIT-result are referred for further examinations.

Digital communication with authorities and the health care system is mandatory in Denmark. Disabled citizens may be exempt from digital communication; they receive postal mail from authorities using a remote printing system [20]. As of March 2018, the proportion of 45-74-year-old citizens exempt from digital communication was 7.9% [21]. In Denmark health care, including screening and any subsequent treatment, is tax-funded and equally accessible to all citizens [22].

### Study design

This study was based on The Lower Educational Attainment Decision aid (LEAD) trial [19] which was a randomised controlled trial conducted among citizens with lower educational attainment, defined as level 1–2 according to the International Standard Classification of Education (ISCED 2011) [17]. This study was a parallel randomised controlled trial including all citizens eligible for CRC screening in the Central Denmark Region regardless of educational attainment. Thus, this study differs from the LEAD protocol by including all citizens eligible for screening and not only those with lower educational attainment. Further, there was a historic cohort included in the original study to assess the Hawthorne effect of receiving the baseline questionnaire hence knowing to be part of a research project. In the original study the Hawthorne effect was similar in intervention and control groups and therefore unlikely to have affected the differences in scores between the groups [18]. Thus, only intervention and control groups were included in this study. Otherwise the research protocol was followed.

A link to the baseline questionnaire was sent via digital mail prior to invitation to take up screening. Subsequently, all baseline questionnaire respondents were simultaneously randomised into intervention and control groups in a 1:1 ratio using a computer-generated algorithm for randomization based on a simple randomization procedure randomly assigning participant ID numbers to intervention or control group. Randomization was conducted by a data manager outside the research group. Participants were not able to change study arm after allocation. Follow-up questionnaires were sent out to all citizens in the study population 90 days after screening invitations had been sent out.

Non-respondents to questionnaires received a digital reminder after two weeks. After four weeks, all non-respondents received a telephone call, offering to fill out the questionnaire orally, thereby trying to increase recruitment among hard-to-reach citizens [19].

All questionnaires were distributed using the secure email platform used for mandatory digital communication with the Danish authorities and health care system.

### Participants

A random sample of 10,030 citizens aged 53–74 years due to be invited to take up CRC screening was identified by the Danish Health Data Authority from the Danish Civil Registration System [23]. Those aged 50–52 years were excluded because they would have already been invited to CRC screening. Those who returned a stool sample within 45 days were excluded.

### Intervention

Those randomised to the intervention group, who did not return a stool sample within 45 days, received a link for the decision aid in a separate digital mail following the screening reminder. The development of the decision aid was based on a framework for the development of decision aids which is based on the International Patient Decision Aid Standard collaboration instrument (IPDASi) [10, 24], and has been described elsewhere [16].

In short, the decision aid information about CRC and CRC screening was presented, focusing on the benefits and harms of screening participation in a clear, balanced way. Specifically, information on CRC incidence, mortality and morbidity was provided. Likewise, how to take up CRC screening using the FIT-method and the effects of this procedure were described followed by a description of the colonoscopy examination. Lastly, possible benefits such as increased survival and decreased morbidity were presented along with possible harms, such as the risk of false positive and false negative results and colonoscopy complications.

In order to embrace different information needs and different levels of understanding, all information was presented primarily in figures and charts with a minimum of text, but with the possibility of opening up pop-up boxes with more information and a "read-more"-function, enabling interested users to access this more detailed information.

Information was presented in 16 steps. A values clarification question was provided in each step, summarised at the end of the decision aid, encouraging the readers to reflect on the information received about whether to participate in screening, and thereby guiding them in making a decision based on their own values. The decision aid is accessible in Danish upon request to the authors.

The control group received no further material after invitation and one standard reminder 45 days after initial invitation.

### Outcomes and background data

The primary outcomes of this trial were the components of informed choice, which is often assessed using three dimensions: knowledge about the options to choose from, attitudes towards the options, and actual behaviour [4, 5]. No single measure for informed choice was made, since we chose to assess knowledge and attitudes as continuous measures instead of dichotomising them. This method was chosen since the definition of an arbitrary cut-off in a continuous scale has been doubted [25].

The outcomes were assessed in questionnaires. At baseline and follow-up, knowledge was assessed using a seven-item scale developed and validated by the research group based on a previous study of citizens’ information needs [26] and literature search. The scale was confirmed unidimensional in factor analyses and had reasonable internal consistency (Cronbach’s α: 0.6; scores range 0–7).

Attitudes were assessed at baseline and follow-up using an existing four item scale developed by Marteau et al. [27]. The scale was translated into Danish by the research group, using a forward-backward method [28] and had a good internal consistency (Cronbach’s α: 0.7; scores range 4–28).

Screening uptake was defined as having returned a stool sample within 90 days after the first screening invitation had been sent out. This definition is in accordance with the Danish Colorectal Cancer Screening Database [29], from which the data were collected.

Secondary outcomes were decisional conflict and stated use of the decision aid. Decisional conflict was assessed at follow-up using an existing 16-item scale [30] which had previously been translated into and validated in Danish, and also had good internal consistency (Cronbach’s α: 0.95; scores range 0–100). At follow-up, citizens in the intervention group were asked whether they had used the decision aid (yes or no).

Background data were linked from Statistics Denmark [31] upon completion of data collection. Ethnicity was categorised as Danish, Western Immigrant and non-Western immigrant, according to the definition by Statistics Denmark. Marital status was dichotomised into married/cohabitant and single/living alone. Income was categorised into three groups based on the dataset tertiles; <€30,000, €30,000–€43,000 and ≥€43,000. Educational attainment was categorised into lower (≤10 years), medium (10–15 years) and higher (>15 years) educational attainment, according to the ISCED 2011 [17]. Occupation was categorised into Self-employed/Chief executive; Employed; Not employed/welfare benefits; Retired and other. Lastly, population density was categorised into three groups (densely populated, intermediate density and thinly populated areas) according to definitions from Statistics Denmark.

### Power calculations

As described previously [19], 200 citizens needed to be included in each final group, requiring a total study population of 10,000 citizens to be initially approached. This number was based on power calculations considering an 80% statistical power and a 5% significance level being able to detect an expected difference of 14 percentage points in attitudes between the intervention and control groups among lower educational attainment citizens only. Including all citizens regardless of educational attainment provided an increase in statistical power in the un-stratified analyses.

### Randomisation

Respondents to the baseline questionnaire were simultaneously randomised into intervention or control group. Allocation was based on participants’ record-ID numbers using a computer-generated algorithm for randomization based on a simple randomization procedure. The algorithm was generated by an administrator of the REDCap (Research Electronic Data Capture) software [32], which was otherwise not attached to the study. No blinding was used.

### Statistical methods

Comparisons were made between the intervention and control groups as intention to treat analyses.

Differences between the background characteristics of the groups were assessed using frequency tables. Differences were tested using chi^2^-test for categorical variables, and the Kruskal Wallis non-parametric test for the continuous age variable.

In this analysis, the overall sample effect was assessed, as well as the effects among citizens with medium and higher educational attainment. The effect of the decision aid among citizens with lower educational attainment has been assessed previously [18]; data were presented for comparison purposes only.

The effects of the decision aid on the outcomes were assessed using linear regression models for continuous and ordinal outcomes (knowledge, attitudes and decisional conflict) that resembled normal distributions, as checked by histograms and qq-plots. For the dichotomous outcome (uptake), logistic regression analysis was conducted. Additionally, analyses stratified by educational attainment were conducted, and lastly, the Wald test was conducted in order to test for effect modification by educational attainment. Due to the randomised controlled design, no adjustments were made.

The proportion of citizens in the intervention group stating that they had used the link for the decision aid was assessed using frequency tables. The proportion was estimated overall and stratified by educational attainment. Proportion differences between groups were tested using the two-sample z-test. The p-values were not adjusted for multiple comparisons.

Lastly, per protocol analyses were conducted by comparing outcomes among stated decision aid users in the intervention group to outcomes in the control group to test the effect of using the decision aid as compared to the intention to treat analyses which tested the effect of receiving it. Linear and logistic regression models were applied.

### Ethics statement

According to the Consolidation Act on Research Ethics Review of Health Research Projects, Consolidation Act number 1083 of 15 September 2017 section 14 (2) notification of questionnaire surveys and medical database research projects to the research ethics committee system is only required if the project involves human biological material. Therefore, this study was conducted without an approval from the committees. The participants gave informed consent to have their data used in research when filling in the first questionnaire. Statistics Denmark linked questionnaire data, data from medical databases, and data on socioeconomic status and anonymized data before we accessed them on a secure research server.

The collection of data from questionnaires and registries was permitted by The Danish Data Protection Agency (J.no.: 2012-58-006 / Case no.: 1-16-02-94-16) and the Danish Patient Safety Authorities (J.no.: 3-313-1729-1).

The trial has been registered in ClinicalTrials.gov (NCT03253888) on the 17^th^ of August 2017 (https://clinicaltrials.gov/ct2/show/NCT03253822).

## Results

### Population characteristics

**Fig 1:**
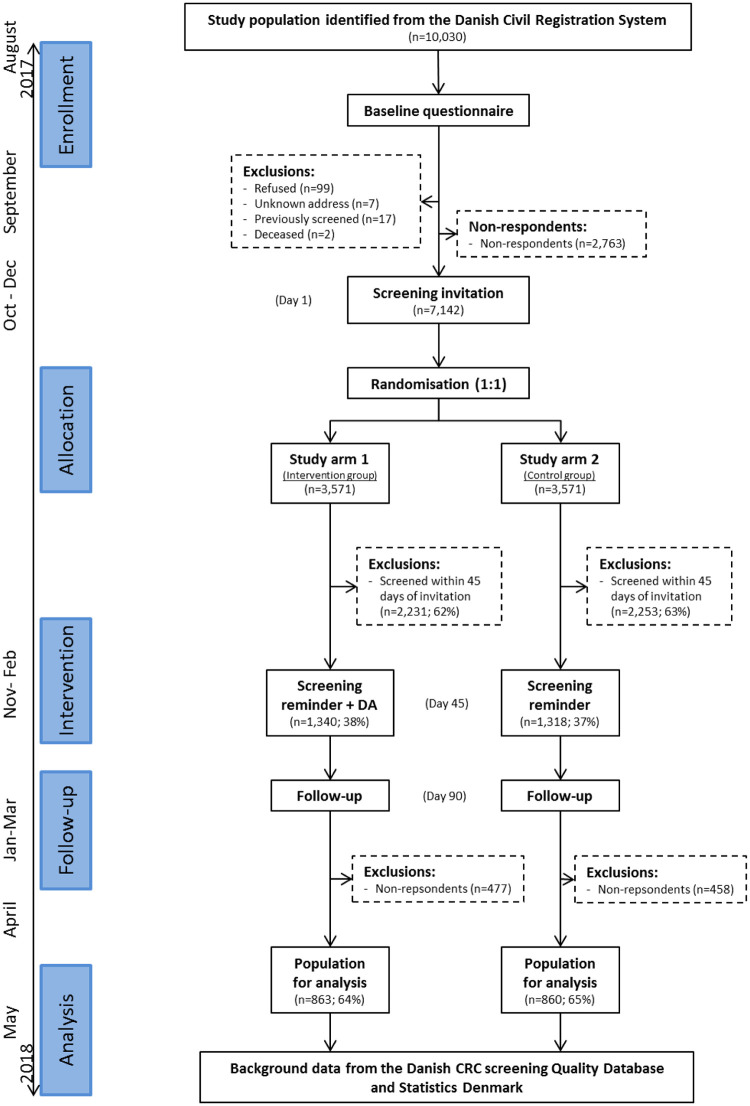
Flow of study population in the trial. CDR: The Central Denmark Region; EA: Educational attainment.

A total of 7,142 citizens (71.2%) filled in the baseline questionnaire. Seven hundred and seven were reached by telephone and a total of 540 completed the questionnaire by telephone. All baseline respondents were subsequently randomised into the intervention and control groups. Of these citizens 4,484 (62.8%) returned a stool sample within 45 days of the invitations being sent out, leaving 1,340 and 1,318 citizens in the intervention and control groups, respectively. Totals of 863 (64%) and 860 (65%) of these citizens completed the follow-up questionnaire, and thereby comprised the study population (Fig 1).

Respondents at baseline and follow-up (comprising the intervention and control groups) were more often of Danish ethnicity, married or cohabitant, of younger age, had medium educational attainment, were employed and had higher income as compared to non-respondents (Table 1). Observed differences between intervention and control groups were not considered clinical relevant.

**Table 1:** Background characteristics.

	Intervention	Control	Non-respondents ^†^
	(n = 863)	(n = 860)	(n = 8,307)
	N (%)	N (%)	N (%)
**Gender**			
Male	425 (49)	446 (52)	3,871 (47)
Female	438 (51)	414 (48)	4,436 (53)
**Age**			
Mean (CI)	62.5 (62.1;62.9)	62.5 (62.1;62.9)	63.9 (63.7;64.0)
53–59	356 (41)	362 (42)	2,753 (33)
60–64	217 (25)	219 (25)	1,920 (23)
65–69	159 (18)	146 (17)	1,886 (23)
70–74	131 (15)	133 (15)	1,748 (21)
**Ethnicity**			
Danish	827 (96)	829 (96)	7,809 (94)
Western immigrant	18 (2)	18 (2)	210 (3)
Non-Western immigrant	17 (2)	13 (2)	275 (3)
**Marital status**			
Married/cohabitant	664 (77)	633 (74)	5,876 (71)
Single	198 (23)	227 (26)	2,418 (29)
**Income**			
< €30,000	230 (27)	227 (26)	2,899 (35)
€30,000-€43,000	273 (32)	277 (32)	2,635 (32)
≥ €43,000	360 (42)	356 (41)	2,773 (33)
**Education**			
≤10 years	173 (20)	166 (20)	2,363 (29)
10–15 years	610 (71)	613 (72)	5,223 (64)
>15 years	71 (8)	68 (8)	527 (7)
**Occupation**			
Self-employed/Chief executive	73 (8)	72 (8)	535 (6)
Employed	420 (49)	433 (50)	3,185 (38)
Not employed/welfare benefits	28 (3)	30 (3)	327 (4)
Retired	333 (39)	310 (36)	4,130 (50)
Other	8 (1)	15 (2)	125 (2)
**Population area**			
Densely populated	174 (20)	168 (20)	1,732 (21)
Intermediate density	245 (28)	256 (30)	2,406 (29)
Thinly populated	444 (51)	436 (51)	4,169 (50)

^†^ Non-respondents in baseline AND/OR follow-up.

No differences were detected between intervention and control groups (p>0.05 for all variables).

Respondents (intervention and control groups) differ from non-respondents in all variables (p<0.01) except for population density, which is similar across groups (p = 0.63).

Out of the 1,723 respondents, 283 were reached by telephone, and completed the questionnaires orally. This group comprised citizens with lower income and education, who were more often retired and living alone in more thinly populated areas. Also the respondents reached by telephone were more likely not to take up screening (n = 226/283; 80%) (data not shown).

### Components of informed choice

The general level of knowledge at baseline was high in both intervention and control groups, reaching 5.05 (95% confidence interval 4.93;5.16) and 5.13 (5.02;5.24), respectively. Likewise, both groups had mainly positive attitudes towards screening at baseline, scoring 20.2 (19.8;20.5) in the intervention group and 20.4 (20.1;20.7) in the control group on the attitudes scale (Table 2).

**Table 2:** General baseline levels of knowledge and attitudes by educational attainment.

	Baseline score			
	Intervention		Control	
**Knowledge**	N	Mean (CI)	N	Mean (CI)
Educational attainment				
≤10 years	*171*	*4*.*76 (4*.*50;5*.*02)*	*164*	*4*.*56 (4*.*29;4*.*84)*
10–15 years	608	5.04 (4.91;5.18)	612	5.24 (5.11;5.36)
>15 years	71	5.76 (5.45;6.07)	67	5.57 (5.24;5.89)
All	850	5.05 (4.93;5.16)	843	5.13 (5.02;5.24)
**Attitudes**				
Educational attainment				
≤10 years	*165*	*19*.*8 (19*.*0;20*.*6)*	*155*	*19*.*9 (19*.*1;20*.*8)*
10–15 years	595	20.2 (19.8;20.6)	602	20.6 (20.2;21.0)
>15 years	70	20.6 (19.5;21.6)	65	19.5 (18.2;20.7)
All	830	20.2 (19.8;20.5)	822	20.4 (20.1;20.7)

Results for citizens with lower educational attainment have been published [18].

In both groups and across all educational attainment levels, a slight increase in knowledge was observed from baseline to follow-up, estimated at 0.14 (-0.19;0.47) points among citizens in the intervention group with higher educational attainment to 0.45 (0.33;0.57) among citizens with medium educational attainment in the intervention group. The mean changes in knowledge between the intervention and control groups were close to zero across all educational levels. The overall difference was 0.09 (-0.05;0.24) (Table 3).

**Table 3:** Decision aid effectiveness on knowledge, attitudes, uptake and decisional conflict by educational attainment.

	**Scale score difference (Baseline to follow-up)**		
	**Intervention**	**Control**	**Comparison**
**Knowledge**	Mean (CI)	Mean (CI)	Mean difference (CI) ^†^
Educational attainment			
≤10 years	*0*.*48 (0*.*21;0*.*75)*	*0*.*48 (0*.*21;0*.*74)*	*0*.*00 (-0*.*38;0*.*38)*
10–15 years	0.45 (0.33;0.57)	0.30 (0.18;0.42)	0.15 (-0.02;0.32)
>15 years	0.14 (-0.19;0.47)	0.37 (0.13;0.62)	-0.23 (-0.64;0.18)
All	0.44 (0.33;0.54)	0.34 (0.24;0.45)	0.09 (-0.05;0.24)
p_interaction_^§^			0.3238
**Attitude**	Mean (CI)	Mean (CI)	Mean difference (CI) ^†^
Educational attainment			
≤10 years	*0*.*49 (-0*.*28;1*.*26)*	*-0*.*22 (-1*.*01;0*.*56)*	*0*.*72 (-0*.*38;1*.*81)*
10–15 years	0.75 (0.35;1.15)	0.28 (-0.09;0.64)	0.48 (-0.06;1.01)
>15 years	-0.32 (-1.40;0.76)	0.06 (-0.91;1.03)	-0.38 (-1.83;1.06)
All	0.62 (0.28;0.95)	0.16 (-0.15;0.47)	0.45 (-0.00;0.91)
p_interaction_^§^			0.5179
	**Proportion taking up screening**		
	**Intervention**	**Control**	**Comparison**
**Uptake**	% (CI)	% (CI)	RD (uptake) ^‡^
Educational attainment			
≤10 years	*34*.*7 (27*.*9;42*.*1)*	*27*.*1 (20*.*9;34*.*4)*	*7*.*6% (-2*.*2;17*.*4)*
10–15 years	43.8 (39.9;47.7)	36.2 (32.5;40.1)	7.6% (-2.1;13.0)
>15 years	45.1 (33.9;56.8)	27.9 (0.19;0.40)	**17.1% (1.4;32.9)**
All	42.1 (38.8;45.4)	34.1 (31.0;37.3)	**8.0% (3.4;12.6)**
p_interaction_^§^			0.5219
	Scale score at follow-up		
	Intervention	**Control**	**Comparison**
***Decisional conflict***	Mean (CI)	Mean (CI)	Mean difference (CI) ^†^
Educational attainment			
≤10 years	*29*.*7 (27*.*4;32*.*0)*	*33*.*2 (30*.*8;35*.*6)*	***-3*.*54 (-6*.*87;-0*.*20)***
10–15 years	32.1 (30.8;33.3)	33.2 (32.0;34.4)	-1.16 (-2.92;0.60)
>15 years	31.1 (26.9;35.3)	32.9 (29.5;36.4)	-1.86 (-7.27;3.55)
All	31.5 (30.4;32.5)	33.2 (32.1;34.2)	**-1.69 (-3.18;-0.20)**
p_interaction_^§^			0.4685

^†^ Linear regression analysis, estimates in bold types are statistically significantly different from 0 (p<0.05).

^‡^ Binary regression model, Risk Difference (RD) estimates in bold types are statistically significantly different from 1 (p<0.05).

^§^ Wald test for interaction.

Results for citizens with lower educational attainment have been published [18].

Attitudes did not change from baseline to follow-up. However, for those with medium educational attainment in the intervention group a small increase (towards being more favourable to CRC screening participation) was observed (0.75 (0.35;1.15)). Likewise, the mean changes between the intervention and control groups were close to zero for all educational groups, with an overall change of 0.45 (-0.00;0.91) points (Table 3).

The overall screening uptake in the intervention group was 42.1% (38.8;45.4). In the control group the overall screening uptake was 34.1% (31.0;37.3). The overall difference between the intervention and control groups was 8.0 percentage points (3.4;12.6), most pronounced among citizens with higher educational attainment where the difference was 17.1 percentage points (1.4;32.9) (Table 3).

### Secondary outcomes

The level of decisional conflict at follow-up was statistically significantly lower in the intervention group as compared to the control group (difference: -1.69 (-3.18;-0.20)). This tendency was also observed among citizens with medium and higher educational attainment levels, but was not statistically significant (Table 3).

Overall, 355 (43.2% (39.8;46.6)) of the respondents in the intervention group stated they had used the decision aid link. Among those who used the link, 50.4% (45.2;55.6) took up screening while the corresponding rate was 35.3% (31.1;39.8) among respondents who did not use the decision aid, corresponding to a 15.1 percentage point difference in uptake (95% CI: 8.3;21.9) (Table 4).

**Table 4:** Decision aid usage and screening uptake by educational attainment.

	Total^a^	Used the DA				
		Yes		No		Difference in uptake rate (CI)^†^
	N	N	% (CI)	N	% (CI)	
Educational attainment						
≤10 years	163	74	45.4 (37.9;53.1)	89	54.6 (46.9;62.1)	
Screened		27	36.5 (26.3;48.1)	26	29.2 (20.7;39.5)	7.3 (-7.2;21.7)
10–15 years	593	250	42.2 (38.2;46.2)	343	57.8 (53.8;61.8)	
Screened		135	54.0 (47.8;60.1)	126	36.7 (31.8;42.0)	**17.3 (9.3;25.3)**
>15 years	66	31	47.0 (35.3;59.0)	35	53.0 (41.0;64.7)	
Screened		17	54.8 (37.1;71.4)	13	37.1 (22.7;54.3)	17.7 (-6.0;41.4)
All	822	355	43.2 (39.8;46.6)	467	56.8 (53.4;60.2)	
Screened	359	179	50.4 (45.2;55.6)	165	35.3 (31.1;39.8)	**15.1 (8.3;21.9)**

^a^ Total number of individuals in the intervention group.

^†^ Two-sample z-test for differences in uptake between the groups.

Respondents with missing values regarding either educational attainment or link usage are omitted from this table.

In per protocol analyses comparing the stated decision aid users to the control group, no difference in knowledge was observed. However, the attitudes were 0.67 (0.08;1.26) points higher (more favourable towards CRC screening), screening uptake was increased by 16.2 percentage points (10.1;22.3) and decisional conflict decreased by 3.96 (5.84;2.07) points in the decision aid users compared with the control group (Table 5).

**Table 5:** Per protocol analyses of decision aid effectiveness on knowledge, attitudes, uptake and decisional conflict.

	DA users	Control	Comparison
	**Scale score difference (Baseline to follow-up)**		
	Mean (CI)	Mean (CI)	Mean difference (CI)^†^
Knowledge	0.45 (0.29;0.61)	0.34 (0.24;0.45)	0.11 (-0.08;0.30)
Attitude	0.83 (0.31;1.35)	0.16 (-0.15;0.47)	**0.67 (0.08;1.26)**
	**Proportion taking up screening**		
	% (CI)	% (CI)	RD (uptake)^‡^
Uptake	50.3 (45.1;55.4)	34.1 (31.0;37.3)	**16.2% (10.1;22.3)**
	**Scale score at follow-up**		
	Mean (CI)	Mean (CI)	Mean difference (CI)^†^
Decisional conflict	29.2 (27.6;30.8)	33.2 (32.1;34.2)	**-3.96 (-5.84;-2.07)**

^†^ Linear regression analysis, estimates in bold types are statistically significantly different from 0 (p<0.05).

^‡^ Binary regression model, Risk Difference (RD) estimates in bold types are statistically significantly different from 1 (p<0.05).

## Discussion

### Main findings

In a randomised controlled trial, we investigated the effect of a web-based decision aid about CRC screening, designed primarily for screening invitees with lower educational attainment levels, on components of informed choice among the general population of Central Denmark Region citizens aged 53–74 years. The decision aid did not affect citizens’ levels of knowledge or their attitudes towards screening. Overall screening uptake was 8 percentage points higher and the level of decisional conflict was slightly lower (1.69 scale points) in the intervention than the control group. The effects for all outcomes were similar across educational attainment levels indicating that the decision aid may be useful in the general screening population.

### Strengths and weaknesses

This study had good internal validity due to low risk of bias and confounding. There was a low risk of selection bias for four reasons; firstly, only citizens who took up screening before the administration of the decision aid were excluded. Secondly, the study benefitted from a high response rate in the questionnaire, with good representation from different educational attainment levels, due to the use of a telephone call instead of the written second questionnaire reminder, although differences between respondents and non-respondents were identified. Thirdly, retrieving background data from validated registries [33] with very few missing values reduced the risk of selection bias, and lastly, concealed randomisation ensured that all citizens had the same probability of being allocated to the intervention and the control groups.

The risk of information bias is also considered small, since the design eliminated the risk of misclassification of the exposure by randomising the citizens into the exposed and not exposed groups. However, the risk of two persons in the same household both invited to be included in the study and subsequently being randomised into different arms cannot be ruled out. Misclassification of the outcome is considered small, since validated scales have been used to classify knowledge, attitudes and decisional conflict, while uptake was classified based on registry data of high validity. However, decision aid usage was self-reported and no data on individual level decision aid usage were systematically collected from the web page, resulting in a small risk of misclassification of this outcome.

The risk of confounding was low, since randomisation ensures equal distribution of known and unknown confounders between the groups. Furthermore, conducting intention-to-treat analyses helps maintain the randomisation, and thereby further reducing the risk of confounding. In the per protocol analysis, however, the decision aid users are self-selected, and hence randomisation has been disturbed. In these analyses, there is a high risk of confounding, considering citizens planning to take up screening are more prone to read additional information material than citizens who already decided not to take up screening.

The trial may suffer from a lack of statistical power, despite the previously conducted power calculations. This is attributable to smaller than expected differences between groups and a smaller than expected study population due to a higher proportion of invited citizens taking up screening within 45 days and lower than expected follow-up questionnaire response rates.

Overall, the external validity is good. The study population is population-based, with a representative sample, selecting citizens based only on age, residence, and screening invitation status, and hence, these citizens are considered representative of the source population of 53–74 year-old Central Denmark region citizens. There may be concerns about whether a web-based decision aid is a suitable way of communication among CRC screening invitees (50–74 years old). However, given that digital communication is mandatory in Denmark [20] most citizens are accustomed to seeking information via the internet. Citizens can be exempt from digital communication, but as of March 2018 the proportion of citizens exempt is only on average 8% among 45–74 year olds [21]. Hence, internet access and skills are considered limited barriers to reach the information in the decision aid in this sample even though it is a barrier to some. Since the populations and management of screening programmes in other Danish regions are like the Central Denmark region population and management, we consider the results of this trial to be generalizable to the rest of Denmark. Further, similar directions of the estimates of effects would be expected in other countries with similar demography and programme delivery for CRC screening.

### Discussion of results and comparison to previous studies

In general we observed a high level of knowledge about CRC screening, which offering the decision aid did not enhance. Previous studies have observed an increase in knowledge among citizens introduced to a self-administered decision aid for CRC screening [6, 7, 11–14]. This may be explained by a true high level of knowledge among Danes eligible for screening but may also reflect an inadequate measurement scale. The scale for measuring knowledge in this study was developed based on citizens’ information needs and the knowledge considered adequate by healthcare professionals to make an informed choice about screening uptake. At baseline 49.2% of respondents scored 6 or 7 out of 7 possible points at baseline indicating that the scale may not differentiate levels of knowledge sufficiently. Further, we observed generally favourable attitude towards CRC screening which was also not affected by the decision aid. Previous studies have observed a change towards less favourable attitudes towards screening after reading a decision aid [6, 7]. Given the favourable attitudes at baseline, a similar change towards less favourable attitudes in the intervention group would be possible, also considering that the decision aid does not promote screening. Finally, screening uptake was statistically significantly increased in the intervention group as compared to the control group. Previous research about screening uptake has been conflicting, with some studies detecting a decrease [6, 11] while increases [15] and no differences [7] have also been detected. In a previous study, an inverted U-shape was observed in the association between educational attainment and screening uptake. That is, those with medium educational attainment more often take up screening than those with lower or higher educational attainments [34]. This tendency was also observed for our control group (uptake rates of 27.1%, 36.2% and 27.9%, respectively). However, offering this decision aid increased the uptake rate among those with higher educational attainment (45.1%) to a comparable level to those with medium educational attainment (43.8%), in the intervention group. Nevertheless, sending out the decision aid in a separate digital mail than the official screening reminder might reflect the effect of a second screening reminder as a co-intervention. Thus, even though the decision aid was sent only a few days after receiving the national reminder, it cannot be ruled out, that the observed effect is partly attributable to the second screening reminder rather than the effect of the decision aid.

As there are no scales validated to differentiate positive and negative attitudes and define "adequate" knowledge with clinical relevance, we chose to focus on the components of informed choice instead of combining them into one measure even though increasing informed choice was the primary end of decision aid. Methods to determine informed choice with greater validity and responsiveness are warranted in future research. However, in both intervention and control group there were high level of knowledge and positive attitudes and since uptake increased in the intervention group, there may be indications that the decision aid increased informed choice. This is in line with previous studies, where informed choice was increased by decision aids [6, 7, 11].

Only 43.2% of citizens in the intervention group stated that they had used the link for the decision aid. It was not possible to gather individual level electronic data regarding decision aid usage via the web page. However, the study population consisted of citizens who did not take up screening within 45 days of receiving the invitation, thereby comprising both those choosing not to take up screening and those who had not got around to sending their sample back; the first sub group being less prone to read additional screening information. Nevertheless, informed decisions about screening are important regardless of whether the decision is to take up screening or not. Therefore, it could be argued that the decision aid should have been provided at the time of invitation instead of at the time of reminder. This decision was made trying to balance the need to support an informed choice with the risk of information overload since they receive the national information leaflet from the Danish health authorities along with the invitation. However, sending out the decision aid at the time of the screening invitations might increase its effectiveness, enabling all invited citizens to make more informed choices about screening, and not just initial non-responders to screening invitations.

Finally, since informed (non)uptake is especially challenged by the home-based procedure in FOBT screening for colorectal cancer, future research may focus on how to further refine self-administered decisions aids and methods to evaluate their effect.

## Conclusions

This study demonstrated high levels of knowledge about CRC and CRC screening and generally favourable attitudes towards CRC screening among citizens eligible for CRC screening, which the decision aid did not enhance. However, the overall uptake rate increased by 8 percentage points indicating that the decision aid may increase informed choice given the increase is among those with high knowledge and favourable attitudes. The decision aid provides a simple intervention supporting users of different educational attainments to access information to greater or lesser degrees of detail, according to their preferences. However, the focus remains on how to further refine self-administered decisions aids and methods to evaluate their effect.

## Supporting information

S1 Checklist CONSORT 2010 checklist of information to include when reporting a randomised trial*. (DOCX)

S1 File (PDF)

S2 File (TXT)
